# High-Fat Mouse Model to Explore the Relationship between Abnormal Lipid Metabolism and Enolase in Pancreatic Cancer

**DOI:** 10.1155/2023/4965223

**Published:** 2023-09-12

**Authors:** Lin Qin, Kai Sun, Li Shi, Yushan Xu, Rongping Zhang

**Affiliations:** ^1^Department of Endocrinology, The First Affiliated Hospital of Kunming Medical University, Kunming, Yunnan 650000, China; ^2^School of Pharmaceutical Science, Kunming Medical University, Kunming, Yunnan 650500, China; ^3^School of Chinese Materia Medica and Yunnan Key Laboratory of Southern Medicine Utilization, Yunnan University of Chinese Medicine, Kunming, Yunnan 650500, China; ^4^Yunnan Province Clinical Research Center for Metabolic diseases, Kunming, Yunnan 650000, China; ^5^Yunnan Clinical Medical Center for Endocrine and Metabolic Diseases, Kunming, Yunnan 650000, China; ^6^Affiliated Hospital of Yunnan University, Qingnian Road, Kunming, Yunnan 650000, China

## Abstract

Malignant tumors have become a major social health problem that seriously threatens human health, among which pancreatic cancer has a high degree of malignancy, difficult diagnosis and treatment, short survival time, and high mortality. More and more attention has been paid to abnormal lipid metabolism as a momentous carcinogenesis mechanism. Here, we explored the relationship between abnormal lipid metabolism, enolase, and pancreatic cancer by clinical data analysis. A high-fat mouse model was constructed, and then, a subcutaneous tumorigenesis mouse model of carcinoma of pancreatic cells and a metastatic neoplasm mouse pattern of pancreatic carcinoma cells injected through the tail vein were constructed to explore whether abnormal lipid metabolism affects the progression of pancreatic cancer in mice. We constructed a high-lipid model of pancreatic carcinoma cell lines and knockdown and overexpressed enolase in pancreatic carcinoma cell lines and investigated whether high lipid regulates epithelial-mesenchymal transition (EMT) by upregulating enolase (ENO), thereby promoting the cells of pancreatic carcinoma to invade and migrate. Triglycerides, total cholesterol, free cholesterin, high-density lipoprotein cholesterol (HDL-C), low-density lipoprotein cholesterol (LDL-C), and neuron-specific enolase (NSE) from pancreatic cancer patients and nonpancreatic cancer patients were tested. The differences in blood lipids between patients with and without pancreatic carcinoma were compared, and the correlation between blood lipids and neuron-specific enolase was analyzed. We confirmed that the serum triglyceride level of pancreatic cancer patients at initial diagnosis is overtopping nonpancreatic cancer patients, and the neuron-specific enolase level of patients with pancreatic carcinoma is better than nonpancreatic carcinoma sufferers. Triglyceride level is positively correlated with neuron-specific enolase level, and serum triglyceride level has predictive value for pancreatic cancer. Hyperlipidemia can promote tumor growth and increase the expression levels of ENO1, ENO2, and ENO3 in subcutaneous tumor formation of pancreatic cancer in mice. Additional hyperlipidemia promoted pancreatic carcinoma metastasis in the lung in mice injected through the tail vein, which confirmed that hyperlipidemia accelerated the process of EMT by increasing the expression of ENO1, ENO2, and ENO3, therefore promoting the pancreatic cancer cell metastasis.

## 1. Introduction

Malignancy has become a major public health issue that seriously threatens human health, among which pancreatic cancer has a high degree of malignancy, difficult diagnosis and treatment, short survival time, and high mortality [1]. Dyslipidemia more and more is considered a significant mechanism of tumorigenesis. The basic test indexes of blood lipid included total cholesterol (TC), low-density lipoprotein cholesterol (LDL-C), high-density lipoprotein cholesterol (HDL-C), and triglyceride (TG) [2]. The metastatic ability of carcinoma cells is strongly linked to lipid metabolic enzymes, In melanoma metastasis, mammary carcinoma, and prostate cancer, the metastatic ability of carcinoma cells is strongly linked to lipid metabolic enzymes [3]. HDL and LDL major cholesterol carriers function through receptor-mediated mechanisms in tumor cells. The effect of HDL cholesterol on cancer is related to its antioxidant and anti-inflammatory properties, and some prospective studies have shown that prediagnosis HDL cholesterol levels are significantly associated with the incidence of breast, prostate, colon, lung, and liver cancers. Serum triglyceride level is significantly increased in patients with colorectal cancer (CRC), and hypertriglyceridemia is positively correlated with the occurrence of colorectal adenoma [4]. Elevated triglyceride can be a colorectal adenoma risk factor for the potential occurrence and development. Enolase (ENO) is an ancient enzyme with a highly conserved structure, including ENO1, ENO2, and ENO3. The active ENO exists as a dimer and consists of two relatively parallel subunits [5]. ENO1 is widely distributed in various tissues, ENO2 is mainly found in neurons, and ENO3 is mainly found in muscle tissues [6]. As a key enzyme in the glycolytic pathway, ENO is related to the prognosis of many such as tumors and other diseases. ENO1 expression is significantly upregulated in all kinds of malignancies, ranging from glioma, nasal laryngeal carcinoma, mammary cancer, and pancreatic cancer to uterine cancer, etc. Studies have found that in pancreatic cancer, the expression of ENO1 is positively correlated with clinical stage and lymphatic metastasis and negatively correlated with survival time [7]. The median survival of patients with high ENO1 expression in pancreatic carcinoma is as short as 8 months, while the median survival time is more than 30 months with low ENO1 expression [8]. ENO1 and related antibodies can reference as underlying biomarkers in the diagnosis and prognosis of tumor. ENO2 is an important tumor marker for the diagnosis, prognosis, and follow-up of lung cancer [9]. The objective was to investigate what is the effect of ENO in abnormal lipid metabolism and how it impacts the progression of carcinoma of pancreas and its mechanism. A basis for individualized diagnosis and therapeutic of pancreatic cancer patients in the future.

The incidence of pancreatic cancer increased in recent years; obesity might be involved, The incidence of pancreatic cancer increased in recent years [10]. Pancreatitis and pancreatic cancer are both pancreatic diseases with common risk factors and pathological features, suggesting a strong correlation between them, which may also be the key to cancer transformation [11]. Acinar-to-ductal metaplasia is observed in chronic pancreatitis. ADM is considered to be a precursor of pancreatic cancer [12]. Oxidative stress and inflammatory response can promote the development of pancreatitis and act together with genetic factors, such as oncogenic KRAS mutation and tumor suppressor gene inactivation, thus initiating and accelerating pancreatic intraepithelial neoplasia [13]. PANIN ultimately leads to pancreatic cancer [14]. Studies have also found that hyperglycemia can increase the incidence rate and sprout of pancreatic cancer in varied ways, and elevated fasting blood glucose can significantly affect the incidence of pancreatic cancer, suggesting that hyperglycemia is closely related to pancreatic cancer [15]. Hyperglycemia is associated with genomic instability by increasing the level of O-GlcNacylation after translation, leading to an imbalance in the nucleotide pool and ultimately to the induction of KRAS mutations, thus becoming an initiating event in pancreatic cancer [16].

Enolase (EC4.2.1.11) is a highly conservative glycosome that catalyzes 2-phospho-D-glyceric acid (2-PGA) to convert to phosphoenolpyruvate (PEP) during glycolysis [17]. Enolase isoenzymes function as homodimers (*αα*, *ββ*, and *γγ*) or heterodimers (*αβ* or *αγ*), catalyzing 2-phosphoglycerate in glycolysis to convert to phosphoenolpyruvate (PEP) [18]. The expression of enolase in mammals is tissue-specific, *α*-enolase (ENO1) is widely found in a variety of tissues, *β*-enolase (ENO3) is mainly expressed in muscle tissues, and *γ*-enO2 is very active in neuronal tissues, so it is also called neuron-specific enolase (NSE) [19]. *γ*-Enolase (ENO2) is the encoding gene located on human chromosome 12. It is composed of two enolase isoenzymes, *γγ* and *αγ* [20]. It is an acid dimer protein with 433 amino acids. By the way, *γ*-enolase also is major in lung cancer diagnosis and prognosis and likely plays a part in predicting chemotherapy response and recurrence in acute leukemia [21]. *γ*-Enolase phosphorylates GSK-3*β* to enhance the activity of Akt and induce cell proliferation, resulting in the increased expression of multiple glycolytic-related genes in acute leukemia cells. Elevated *γ*-enolase expression is also associated with neuroblastoma, cervical cancer, melanoma, renal cell carcinoma, and other diseases [22]. ENO2 serves as a potential target for these tumors [23]. A positive correlation was found by Chang et al. between serum triglyceride extent and the NSE level of ischemic stroke patients [24]. However, there are few studies on the effect of abnormal lipid metabolism on ENO and its further effect on pancreatic carcinoma, which is worthy of further investigation.

## 2. Materials and Methods

### 2.1. Preparation of Animals

This research was supported by the Kunming University of Medical Sciences (Approval number: kmmu2021426).

As laboratory animals, 6-8-week-old male C57 and Panc-02 mice weight 20 g-30 g (*n* = 12, each group), and Panc-02 mouse pancreatic cancer cells were provided by Olubiol (Kunming, Yunnan, China). The mice were housed in an animal facility and handled in accordance with the Guide for the Care and Use of Laboratory Animals of Kunming Medical University Hospital. All mice were housed under ambient conditions (standard humidity and temperature) with a 12 h light/dark cycle. The 7-week-old mice were used for experimentation after an adaptation period of 1 week. All mice were specifically pathogen-free and were maintained under the same environmental conditions without differences in food intake.

### 2.2. The Research Object

All 208 pancreatic carcinoma patients admitted to the First Affiliated Hospital of Kunming Medical University from January 2016 to June 2022 were enrolled as the study group, including 117 males and 91 females. The inclusion criteria of the pancreatic cancer group were as follows: malignant cells of pancreatic cancer were detected by pancreatic cancer surgery or biopsy, which met the diagnostic criteria of pancreatic cancer. Exclusion criteria for the pancreatic cancer group were as follows: age < 18 years and without a pathological diagnosis of pancreatic cancer. The control group included 1350 randomly selected people who underwent physical examination in our hospital during the same period. The exclusion criteria of the control group included those who were younger than 18 years old, had pancreatic occupation on ultrasound, or had a history of pancreatic cancer. There were 662 males and 688 females in the control group. There was no statistical significance in the sex ratio between the two groups (*P* > 0.05).

### 2.3. The Indicators

The indicators used in this study include gender, age, aspartate aminotransferase (AST) levels (IU/L), alanine aminotransferase (ALT) levels (IU/L), serum creatinine (Cre) levels (*μ*mol/L), serum uric acid (UA) levels (*μ*mol /L), fasting venous glucose levels (mmol/L), total cholesterol (TC) levels (mmol/L), free cholesterol (F-CHOL) levels (mmol/L), triglyceride (TG) levels (mmol/L), high-density lipoprotein cholesterol (HDL-C)(mmol/L), low-density lipoprotein cholesterol (LDL-C) (mmol/L), and neuron-specific enolase (NSE) (ng/mL). All indicators were collected from the Clinical Data Management Center of the First Affiliated Hospital of Kunming Medical University.

### 2.4. Detection Method

Roche Cobas 8000 automatic biochemical analyzer was used to measure blood biochemical-related indexes (fasting blood in the morning): total protein (biuret method), blood glucose (hexokinase method), total cholesterol (cholesterol oxidase method), triglyceride (GPO-PAP method), and low-density lipoprotein cholesterol (surfactant clearance method). Tumor markers were detected by the Cobas 6000 instrument. Within two hours, the professional inspectors of the Central Laboratory of the First Affiliated Hospital of Kunming Medical University shall complete the inspection with original reagents according to the standard procedures of the instrument usage instructions and strictly control the quality. An abdominal ultrasound examination was completed in the Imaging Department of the First Affiliated Hospital of Kunming Medical University.

### 2.5. Immunohistochemistry Was Used to Detect Clinical Specimens

The surgical sections of patients with clinical pancreatic cancer were collected for immunohistochemical staining. The specific steps were as follows: (a) baking sheet: put the tissue sheet into a 64°C incubator and bake for 1 h; (b) dewaxing: put the glass slide into xylene I (10 min) and xylene II (10 min); (c) hydration: 100% alcohol I and II (5 min), 100% alcohol (5 min), 95% alcohol (5 min), 80% alcohol (3 min), and 70% alcohol (2 min); rinse with PBS 3 times, 5 min each time; (d) antigen repair: pour citrate buffer into the pressure cooker and boil it, then put the slide in, cover the pot tightly, start the timer when a large number of bubbles emerge from the exhaust valve, boil for 3 minutes, turn off the heat, open the cover after exhaust, and cool to room temperature; rinse with PBS 3 times, 5 min each time; (e) blocking: incubation with 3% H_2_O_2_ water for 20 min at room temperature to inactivate endogenous peroxidase activity; rinse with PBS 3 times, 5 min each time; (f) block: 5% bovine serum albumin V was incubated at 37°C for 30 min; (g) incubation of primary antibody: according to the antibody instructions, the appropriate dilution ratio was selected to dilute the primary antibody with 2% bovine serum albumin V, and the diluted primary antibody was added to the glass slide by drop and placed in the refrigerator at 4°C overnight. In the next day, the primary antibody was placed in the temperature box at 37°C for rewarming for 30 min and then washed with PBS three times, 5 min each time; (h) incubation of secondary antibody: goat anti-rabbit secondary antibody (diluted in PBS, 1 : 500) was added to the tissue block and incubated at 37°C for 30 min and then washed with PBS 3 times, 5 min each time; (i) DAB color development: DAB staining droplets were added to the tissue blocks for staining, and the slides were placed under a microscope to observe the staining. After obvious staining, the staining solution was washed with PBS, and the staining solution was washed with PBS 3 times, 5 min each time; (j) hematoxylin counterstaining: the slide was stained in hematoxylin for 5 min, washed with distilled water, put into alcohol hydrochloric acid solution for differentiation, differentiation for 10-15 s, and put into tap water to return blue for at least 15 min; (k) dehydration: 70% alcohol (2 min), 80% alcohol (3 min), 95% alcohol (5 min), 100% alcohol I (5 min), and 100% alcohol II (5 min); (l) transparent: xylene I (10 min) and xylene II (10 min); (m) seal: seal the film with neutral gum; (n) analysis: for microscopic observation, 5 visual fields were selected to take films and the positive rate was calculated.

### 2.6. Mouse Pancreatic Cancer Cell Panc-02 Cell Culture

#### 2.6.1. Cell Recovery

According to the records, the frozen cells were removed from liquid nitrogen and quickly shaken in a 37°C water bath. After they were dissolved, the cells were quickly brought to the ultraclean workbench. The cells were transferred to a 15 mL centrifuge tube containing 10 mL complete medium, mixed, and centrifuged at 1000 rpm for 5 min at room temperature. The supernatant was poured out and DMEM complete medium was added. After blowing and mixing, the cell suspension was transferred into T-25 culture flask and cultured in an incubator with 5% CO_2_ at 37°C.

#### 2.6.2. Cell Passage

When the cell density reached 80%, carefully absorb and discard the culture medium in the cell culture dish with a pipette gun on the ultraclean workbench, slowly add 3 mL sterile PBS from the edge of the dish with a pipette gun, absorb and discard PBS with a pipette gun, and wash twice. When most of the cells became round and separated from each other, an appropriate amount of complete medium containing fetal bovine serum was added to terminate the digestion. The single cell suspension was made by gently blowing and was centrifuged at 1000 rpm for 5 min, the medium was discarded, and the cells were resuspended by adding a complete medium containing fetal bovine serum. Then, it was divided into culture bottles for further culture and subcultured according to 1 : 3.

#### 2.6.3. Cell Cryopreservation

When the cell density reached 80%, the culture medium in the cell culture dish was carefully sucked and discarded with the pipette gun on the ultraclean workbench, 3 mL sterile PBS was slowly added from the edge of the dish with the pipette gun, the PBS was sucked and discarded with the pipette gun, and the washing was repeated twice. When most of the cells became round and separated from each other, an appropriate amount of DMEM complete medium containing fetal bovine serum was added to terminate the digestion, and the single cell suspension was made by gently blowing and centrifuged at 1000 rpm for 5 min, and the medium was discarded. The cells were resuspended by adding 1 mL of frozen storage solution and transferred to the frozen storage tube and placed in the frozen storage box at -80°C overnight and then transferred to liquid nitrogen for storage.

### 2.7. Blood Samples Were Taken from Mice to Detect Blood Lipids

There are 24 C57 mice (male) aged 6-8 weeks, of which 12 mice were fed with high fat and 12 mice were fed with normal. After 6 weeks, blood samples were collected from the eye socket of mice and placed in heparin anticoagulant tubes for 2 hours. After that, the samples were separated at 3000 rpm/heart for 15 min at 2-8°C. The thawed samples were centrifuged again and then tested for triglyceride, total cholesterol, HDL cholesterol, and LDL cholesterol levels.

### 2.8. Tumor Formation by Subcutaneous Injection

Six mice were randomly selected from 12 mice with hyperlipidemia after 6 weeks of high-fat feeding, 6 mice were randomly selected from 12 mice with normal blood lipids, 6 mice were also randomly selected from 12 mice with normal blood lipids, and 6 mice were also randomly selected from 12 mice with normal blood lipids at 8 weeks. 100 *μ* of PANC-02 mouse pancreatic cancer cells (7 × 10^6^ cells) was subcutaneously injected into C57 mice. We checked whether the cell name corresponds to the group of animals one by one; check whether the cell name corresponds to the group of animals one by one. The air in the syringe should be discharged after the syringe inhales the cells. Pinch the skin at the injection site with your hand, stab the needle into the subcutaneous area and groin observedly, draw back if no blood, and then, advance the cells; hold for 7-10 s, pull out the needle, and locally compress it with a cotton ball or swab for a while. The long and short diameters of the implanted subcutaneous tumors were measured with vernier calipers every 2 days from 3 days after subcutaneous tumor modeling, and the tumor volume was calculated according to the formula *V* = *A* × *B*^2^ × 0.5 (*V* represents the tumor volume, *A* is the long diameter, and *B* is the short diameter). On the 12th day after subcutaneous tumorization, the animals were sacrificed by cervical dislocation, and the tumor pieces were slowly removed with ophthalmic scissors and ophthalmic tweezers and then weighed on an electronic balance. The tumor mass was measured by analytical balance and recorded.

### 2.9. Tumor Formation by Tail Vein Injection

Twelve mice were given a high-fat diet (high-fat group), while the other twelve mice were given a normal diet (control group). Blood lipids were measured after six weeks, and at the 8th week, six mice from each group were randomly selected for tail vein injection with pancreatic cancer cells. 100 *μ*L of PANC-02 mouse pancreatic cancer cells (5 × 10^6^ PANC − 02 cells) was injected into C57 mice through tail vein. Pancreatic cancer cells (PANC-02 cells) may metastasize to the spleen, lung, pancreas, and brain, causing tissue lesions. Body weight was monitored every 3-4 d after 3 days of surgery. An electronic scale was used to record the weight changes of mice. The steps are as follows: step 1: place the electronic scale on a hard and flat surface; step 2: press the “on/off” button, and the scale will be cleared within 3 seconds; and step 3: please place the item to be weighed in the container, and the weight will be displayed on the electronic screen. After the body weight of C57 mice decreased abruptly, metastatic foci may be formed. On the 42nd day after the tail vein injection of pancreatic cancer cells into the model, C57 mice were anesthetized by intraperitoneal injection of 1.5 vol% isoflurane (1 L/min) through a 1 mL syringe according to their body weight. After that, the abdomen was disinfected and the skin was prepared. Lung tissue was removed from mice.

### 2.10. QPCR Detection

Total RNA extraction: cells were mixed with 700 *μ*L of RNA extract, thoroughly blown and mixed, and then stood for 10 min; 140 *μ*L of chloroform was added and thoroughly mixed. Centrifugation at 12000 g for 15 min at 4°C showed that the liquid was divided into three layers, and RNA was retained in the colorless upper aqueous phase. Gently draw the upper aqueous phase into a new EP tube and record the volume of the supernatant. Then, the same volume of 100% isopropanol was added and centrifuged at room temperature for 10 min at 12000 g for 10 min at 4°C. It was observed that more white RNA precipitates were generated at the bottom of the tube. Carefully tilt the tube mouth to discard the supernatant, blot the tube mouth with absorbent paper, add 500 *μ*L 75% ethanol to the precipitation (the amount of ethanol added is half of the supernatant), centrifuge at 7500 g for 5 minutes at 4°C, and make the precipitation adhere to the bottom of the tube. Discard the supernatant, invert the centrifuge tube onto absorbent paper, and blot the remaining liquid with a pipette gun. Blow in a ventilated kitchen for 5 minutes to remove as much residual liquid as possible. Add 60 *μ*L of RNase water to the dried RNA precipitate and leave for 15 minutes to dissolve the RNA completely. Freeze in the refrigerator at -80°C. Reverse transcription: SureScript First-Strand cDNA Synthesis Kit (Xavir, Guangzhou, China) was used. After brief centrifugation, the reaction was carried out in CFX96 real-time quantitative PCR instrument according to the following conditions: predenaturation at 95°C for 1 min, denaturation at 95°C for 20 s, annealing at 55°C for 20 s, and extension at 72°C for 30 s, 40 cycles; and the final extension was made at 72°C for 5 min 4°C. Fluorescence was collected and recorded, amplification curve and dissolution curve were made, and Ct values were read. The primer sequence is as follows: GAPDH F: CCTTCCGTGTTCCTACCCC; GAPDH R: GCCCAAGATGCCCTTCAGT; E-cadherin F: GGGACAAAGAAACAAAGGT; E-cadherin R: GACACGGCATGAGAATAGA; N-cadherin F: CCCCCAAGTCCAACATTTC; N-cadherin R: CCGCCGTTTCATCCATACC; vimentin F: GCAGCCTCTATTCCTCATC; vimentin R: TGCAGTTCTACCTTCTCGT; a-SMA F: TGCCGAGCGTGAGATTGT; a-SMA R: CTTCATGGTGCTGGGTGC; ENO1 F: GGCAACCCTGAAGTCATCCT; ENO1 R: AATCCACCCTCATCACCCAC; ENO2 F: GGATGGGACTGAGAATAAA; ENO2 R: AGCAATGTGGCGATAGAGG; ENO3 F: GGGGGATGAGGGTGGCTTT; and ENO3 R: GGGGTTGGTTACCGTGAGG. Analysis of experimental results: the dissolution curve was smooth with only one large single peak, and the primer specificity was good. The data were available. The Ct values were read, and the relative gene expression was calculated using the 2^−△△Ct^ method. Specifically, the first step was calculated as △Ct = Ct (target gene) − Ct (reference gene). △△CT = △CT (experimental group) − △CT (control group); finally, the 2^−△△CT^ value was calculated as the relative expression level of mRNA.

### 2.11. Western Blotting Detection

#### 2.11.1. Tissue/Cell Protein Extraction

Preparation of RIPA lysate: 1 mL RIPA lysate with 10 *μ*L of 100x protease inhibitors (if phosphorylated antibodies need to be checked to add the corresponding phosphatase inhibitors) on ice for use. Discard the medium, wash the cells with precooled PBS for 3 times, add the corresponding amount of cell lysate, lysate on ice for 10 min, scrape the cells with cells, and transfer to EP tube. Weigh 50-100 mg of tissue and add 500-1000 *μ*L RIPA lysate to the tissue homogenizer and homogenize on ice. Centrifuge the above lysed sample at 16000 g for 15 min at 4°C, take the supernatant, and divide it into 80 *μ*L each.

#### 2.11.2. Determination of Protein Concentration and Denaturation

Determination of protein concentration with BCA protein quantification kit: add 0, 0.25, 0.05, 0.1, 0.2, 0.3, 0.4, and 0.5 mg/mL of the standard volume of 20 *μ*L, after 50 times of sample dilution, add 20 *μ*L of the diluted sample to make 3 rewells, and add 200 *μ*L of BCA working solution (BCA reagent A and B 50 : 1 preparation). The absorbance value was measured at 562 nm after 30 minutes at 37°C. Protein denaturation reserve: take 80 *μ*L protein sample for quantification 3.8-6 *μ*g/*μ*L (concentration < 3.8 *μ*g/*μ*L ≥ 0.5 *μ*g/*μ*L quantitative denaturation according to the lowest), mix with appropriate 5× protein loading buffer and boil in boiling water bath for 10 min, cool to room temperature, and store at -80°C.

#### 2.11.3. SDS-PAGE Electrophoresis

Separation glue: distilled water, 30% acrylamide, 1 M Tris-HCl (pH 8.8), 10% SDS, 10% ammonium persulfate, and TEMED preparation according to the different target protein preparation of different concentrations of glue. Concentrated glue: concentrated glue (5 mL) 3.4 mL of distilled water, 30% acrylamide 0.83 mL, 1 Tris-HCl (pH 6.8) 0.63 mL, 10% SDS 0.05 mL, 10% ammonium persulfate 0.05 mL, and TEMED preparation 0.01 mL. After the glue is prepared, add distilled water liquid seal, discard the distilled water after 30 min, and blot the water with paper. The concentrated glue can be used 10 min after it is prepared. SDS-PAGE electrophoresis: 10 *μ*L sample loading and 80 V stable pressure electrophoresis for about 30 min; when the protein sample is to the separation glue concentrated glue interface, change to 120 V stable pressure electrophoresis; when bromophenol blue run to the bottom, about 90 minutes, take out the gel.

#### 2.11.4. Membrane Transfer

The PVDF membrane should be soaked in methanol for 5 minutes and then balanced in the membrane transfer buffer for 15 minutes. The membrane was transferred to the electroconverter at 4°C and 300 mA for 1 h. Block: remove PVDF membrane and rinse with TBST once, cut the excess membrane on the edge, and block in 5% BSA at 37°C for 30 min (or overnight at 4°C).

### 2.12. Statistical Analysis

SPSS20.0 software was used for data processing. The normal distribution of measurement data was expressed as mean ± standard deviation (X ± S), and *t*-test was used for comparison between the two groups. The receiver operating characteristic curve (ROC curve) was used to evaluate the prognostic value. *P* < 0.05 was considered statistically significant.

## 3. Results

### 3.1. Expression and Correlation Analysis of Triglyceride and NSE in Pancreatic Cancer Patients

**Figure 1:**
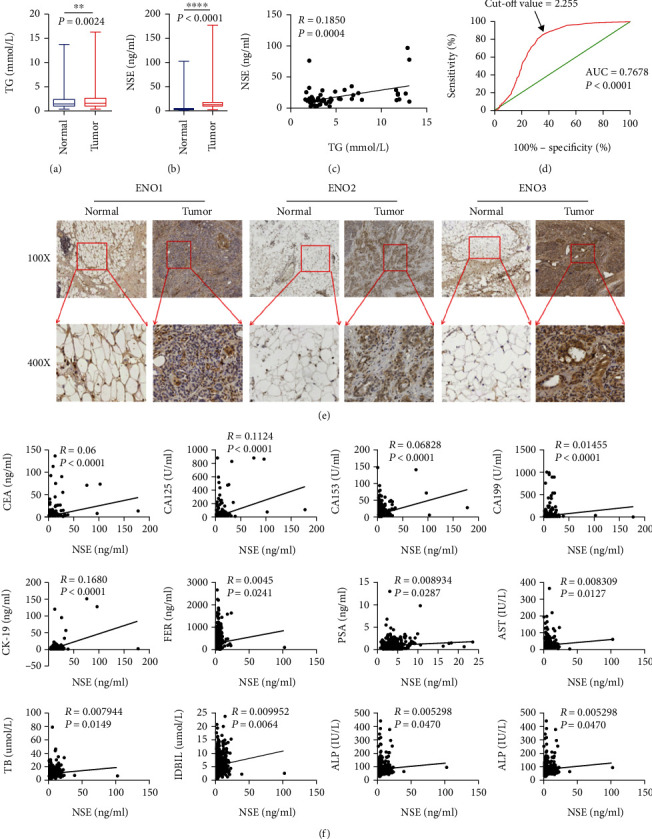
Clinical data analysis of pancreatic cancer patients and control group. (a–c) Expression and correlation analysis of triglyceride and NSE in pancreatic cancer patients. (d) ROC curve was used to evaluate the predictive value of triglyceride in pancreatic cancer. (e) Immunohistochemistry was used to detect the expression of ENO1, ENO2, and ENO3 in clinical tissues. (f) Analysis of neuron-specific enolase (NSE) and tumor-related indicators.

The comparison of clinical data between pancreatic cancer patients and the control group showed that TG and NSE of pancreatic cancer patients were higher than the control group, and there was a significant positive correlation between triglyceride and neuron-specific enolase (NSE) (*P* = 0.0004) (Figures 1(a)–1(c)). ROC curve evaluation showed that triglyceride had a certain predictive value in pancreatic cancer (AUC = 0.7678 > 0.7). AUC > 0.7 is considered to have predictive value; AUC < 0.7 has no predictive value (Figure 1(d)). By analyzing the clinical indicators of pancreatic cancer patients and the control group and exploring the relationship between abnormal lipid metabolism, enolase, and pancreatic cancer, we drew the following conclusions: the triglyceride level of pancreatic cancer patients at initial diagnosis was higher than that of the control group. The level of NSE in pancreatic cancer patients was higher than the control group (Figure 1(e)). Triglyceride level was positively correlated with NSE. Human growth hormone (HGH), ferritin (FER), and pepsinogen 1 were correlated with triglyceride (pepsinogen 1), total bile acid (TBA), gamma-glutamyl transpeptidase (GGT), cholinesterase (PChE), urea nitrogen (BUN), blood uric acid UA, high-density lipoprotein cholesterol (HDL-C), low-density lipoprotein cholesterol (LDL-C); CEA, CA125, CA153, CA199, CK-19, ferritin (FER), prostate specific antigen (PSA), aspartate aminotransferase (AST), total bilirubin (TB), indirect bilirubin (IDBIL), and alkaline phosphatase (ALP) were correlated with NSE (Figure 1(f)).

### 3.2. Identification of Mouse Hyperlipidemia Model

**Figure 2:**
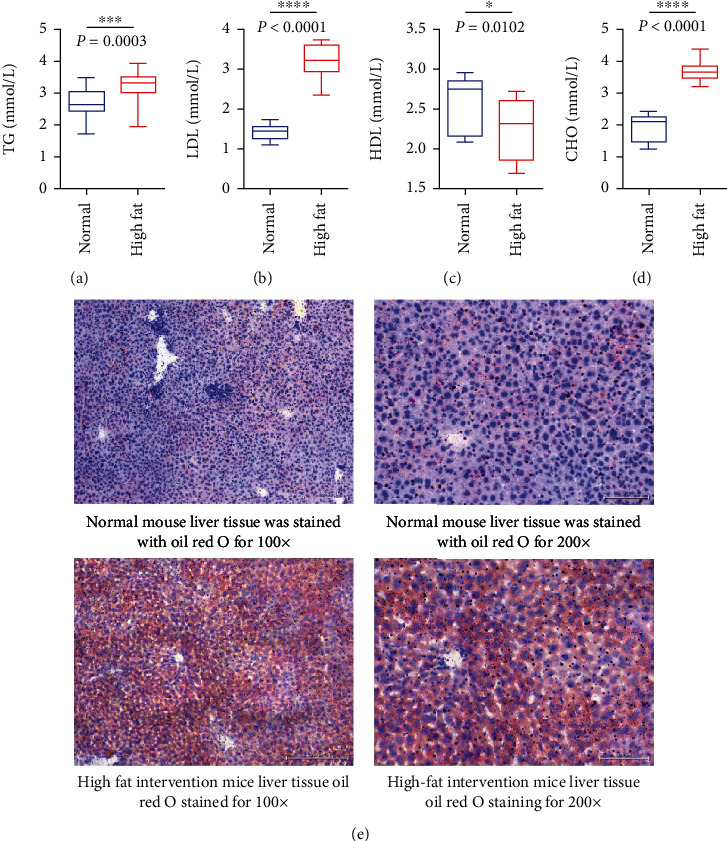
Establishment and identification of mouse hyperlipidemia model. (a) The expression of triglyceride TG in the four blood lipids of mice. (b) The expression of low-density lipoprotein cholesterol and LDL in the four blood lipids of mice. (c) The expression of high-density lipoprotein cholesterol HDL in the four blood lipids of mice. (d) The expression of total cholesterol CHO in the four items of blood lipid detected by total cholesterol CHO in mice. (e) The results of oil Red O staining in liver tissue of mice. The results showed that the fat content in the liver tissue of mice in the high-fat feeding group was significantly higher than that in the ordinary feeding group.

C57 mice in the high-fat group were fed with a high-fat diet, while C57 mice in the normal group were fed with an ordinary diet. After 6 weeks, four blood lipids were detected by a biochemical analyzer. The results of oil Red O staining in liver tissue showed that the hyperlipidemia mouse model was successfully established. Four blood lipids (triglyceride (TG), low-density lipoprotein cholesterol (LDL), high-density lipoprotein cholesterol (HDL), and total cholesterol (CHO)) in mice were detected (Figures 2(a)–2(d)). Compared with the mice in the high-fat feeding group, triglyceride (TG) increased, low-density lipoprotein cholesterol (LDL) increased, high-density lipoprotein cholesterol (HDL) decreased, and total cholesterol (CHO) increased, all with statistical significance. The results of oil Red O staining of mouse liver tissue showed that the fat content of liver tissue in the high-fat feeding group was significantly higher than that in the ordinary feeding group (Figure 2(e)).

### 3.3. The Construction of Pancreatic Cancer Subcutaneous Tumor Animal Model

**Figure 3:**
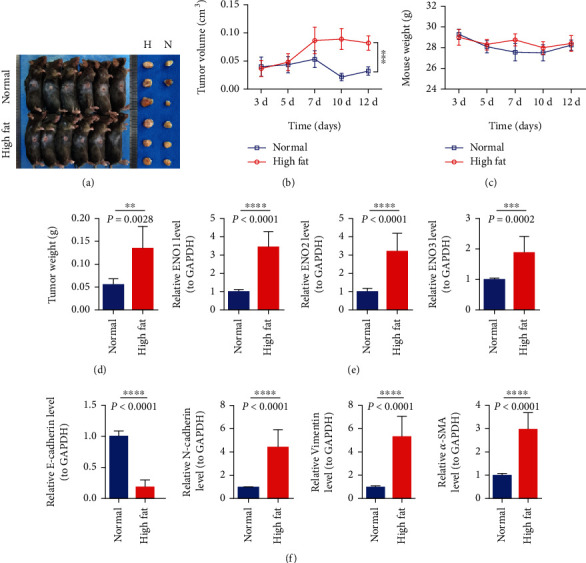
To construct a subcutaneous tumor animal model of pancreatic cancer. (a) Individual and tumor differences in mice; the tumors in the high-fat group were relatively larger. (b) Differences in tumor volume in mice. (c) Body weight growth curves of mice inoculated with tumor. (d) Tumor weight statistics after in vitro. (e) ENO1/ENO2/ENO3 indexes related to tumor enolase in mouse subcutaneous tumorigenesis test. (f) Expression of EMT-related genes E-cadherin/N-cadherin/vimentin/*α*-SMA. ^∗∗∗^ represents *P* < 0.001, and ^∗∗∗∗^ represents *P* < 0.0001.

Mice in the high-fat group and control group were injected with PANC-02 cells subcutaneously to establish the animal model of pancreatic cancer tumorigenesis (Figure 3(a)). The curve of tumor volume change in mice showed a gradual upward trend; the volume of the control group increased after decreasing (Figure 3(b)). The curve of weight change of mice after subcutaneous tumor formation showed that the weight of the high-fat group showed a trend of first increasing and then decreasing (Figure 3(c)), while the weight of the control group gradually decreased. The tumor growth curve (Figure 3(d)) showed that compared with the control group, the tumors in the high-fat group grew relatively faster, and the difference between the two groups was statistically significant.

### 3.4. Expression of Enolase-Related Indexes and EMT-Related Genes in Mouse Subcutaneous Tumorigenesis

**Figure 4:**
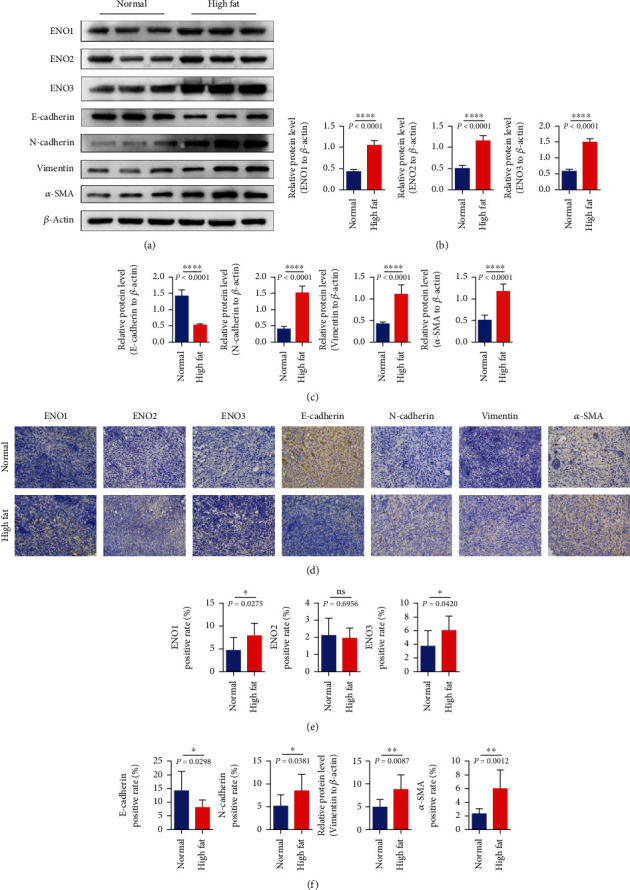
Indexes related to tumor enolase in subcutaneous tumorigenesis of mice. (a–c) The expression of ENO1/ENO2/ENO3 and EMT-related genes E-cadherin/N-cadherin/vimentin/*α*-SMA detected by western blotting in each group of mouse subcutaneous tumor-forming experiment. (d) The expressions of ENO1/ENO2/ENO3 and EMT-related genes E-cadherin/N-cadherin/vimentin/*α*-SMA were detected by immunohistochemistry. Ns represents no statistical significance, ^∗^ represents *P* < 0.05, and ^∗∗^ represents *P* < 0.01.

QPCR test declares that enolase-related indicators ENO1/ENO2/ENO3 in the high-fat group were significantly higher than those in the control group (Figure 3(e)).The expression of EMT-related genes N-cadherin, vimentin, and *α*-SMA in the high-fat group was significantly higher than that in the control group, and the expression of E-cadherin in the high-fat group was significantly lower than that in the control group (Figure 3(f)). These results suggested that the enolase ENO1/ENO2/ENO3 of subcutaneous tumor in the high-fat group increased and promoted the process of EMT (Figure 4).

### 3.5. The Animal Model of Pancreatic Cancer Tail Vein Tumor

**Figure 5:**
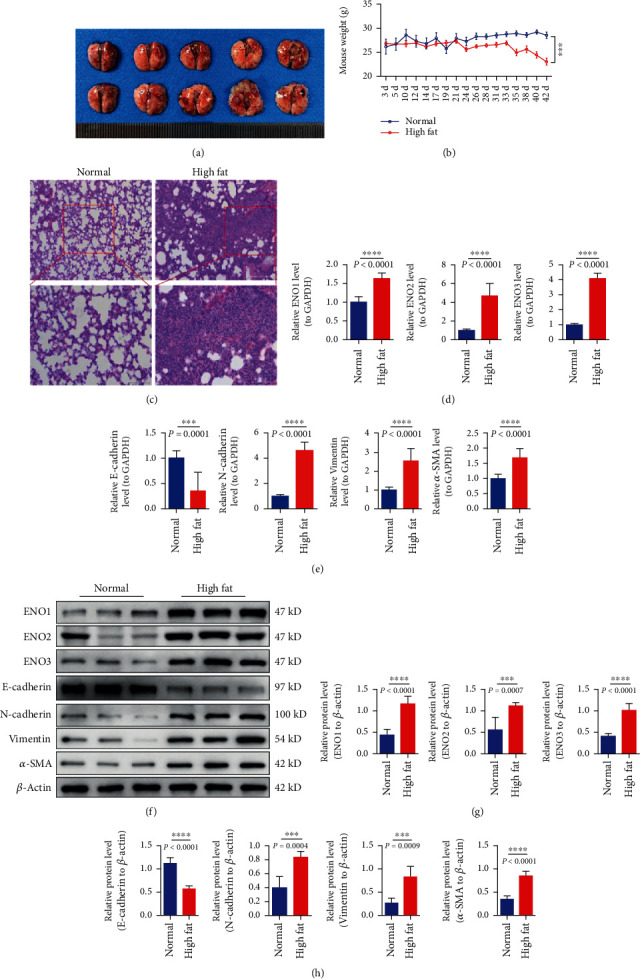
(a) The pulmonary metastatic nodule picture of the lungs. (b) The body weight monitoring map. (c) The HE staining in each group of the tail vein injection. (d, e) QPCR was used to detect the expression of enolase-related indicators ENO1/ENO2/ENO3 and EMT-related genes E-cadherin/N-cadherin/vimentin/*α*-SMA in the lungs of each group after tail vein injection. (f–h) Western blotting was used to detect the expression of enolase-related indicators ENO1/ENO2/ENO3 and EMT-related genes E-cadherin/N-cadherin/vimentin/*α*-SMA in the lungs of mice injected through tail vein. ^∗∗∗^ represents *P* < 0.001 and ^∗∗∗∗^ represents *P* < 0.0001.

Pancreatic cancer cells were injected into the tail vein of high-fat mice and control mice. It was confirmed that hyperlipidemias accelerated the process of EMT and promoted the metastasis of pancreatic cancer cells by stimulating enolase-related indicators ENO1/ENO2/ENO3. The hyperlipidemia model was established in C57 mice. PANC-02 pancreatic cancer cells were injected into the tail vein of both the high-fat group and control group, and the experiment was terminated 42 days later. Lung metastatic nodules of pancreatic cancer cells were observed in mice. The lung was removed and the metastatic nodules of lung tissue were detected. The lung physiology figure showed that the number and volume of metastatic nodules in the high-fat group were larger than those in the control group (Figure 5(a)).

The curve of weight change (Figure 5(b)) showed that the weight of the high-fat group increased first and then decreased, while the weight of the control group gradually increased, and the weight of the high-fat group was significantly lower than that of the control group after 21 days, and the difference was obvious. HE staining showed that the control group had normal lung tissue structure, intact alveoli, no edema, inflammatory cell infiltration, and no tumor cell metastasis. In the high-fat group, the alveolar structure of lung tissue was incomplete, and a large number of tumor cells could be seen metastasis. These results suggest that hyperlipidemia may enhance the ability of pancreatic cancer cells to metastasize to the lung (Figure 5(c)). We detect the expression of enolase-related indicators ENO1/ENO2/ENO3 and EMT markers in lung tumor tissues, and further explore the effect of hyperlipidemia on enolase activation and metastasis in pancreatic cancer cells at the molecular level. QRT-PCR results showed (Figure 5(d)) that enolase-related indicators ENO1/ENO2/ENO3 in the high-fat group were significantly higher than those in the control group. The expression of EMT-related genes N-cadherin, vimentin, and *α*-SMA in the high-fat group was significantly higher than that in the control group, and the expression of E-cadherin in the high-fat group was significantly lower than that in the control group. These results suggested that hyperlipidemia increased enolase expression and accelerated the process of EMT. Western blotting results showed (Figure 5(f)) that enolase-related indicators ENO1/ENO2/ENO3 in the high-fat group were significantly higher than those in the control group. The expression of EMT-related genes N-cadherin, vimentin, and *α*-SMA in the high-fat group was significantly higher than that in the control group, and the expression of E-cadherin in the high-fat group was significantly lower than that in the control group (Figures 5(g) and 5(h)). The results were consistent with those of qPCR. These results suggest that hyperlipidemia increases enolase and promotes EMT.

### 3.6. Upregulation of Enolase Can Accelerate the Process of EMT and Aggravate the Malignant Behavior of Pancreatic Cancer Cells

**Figure 6:**
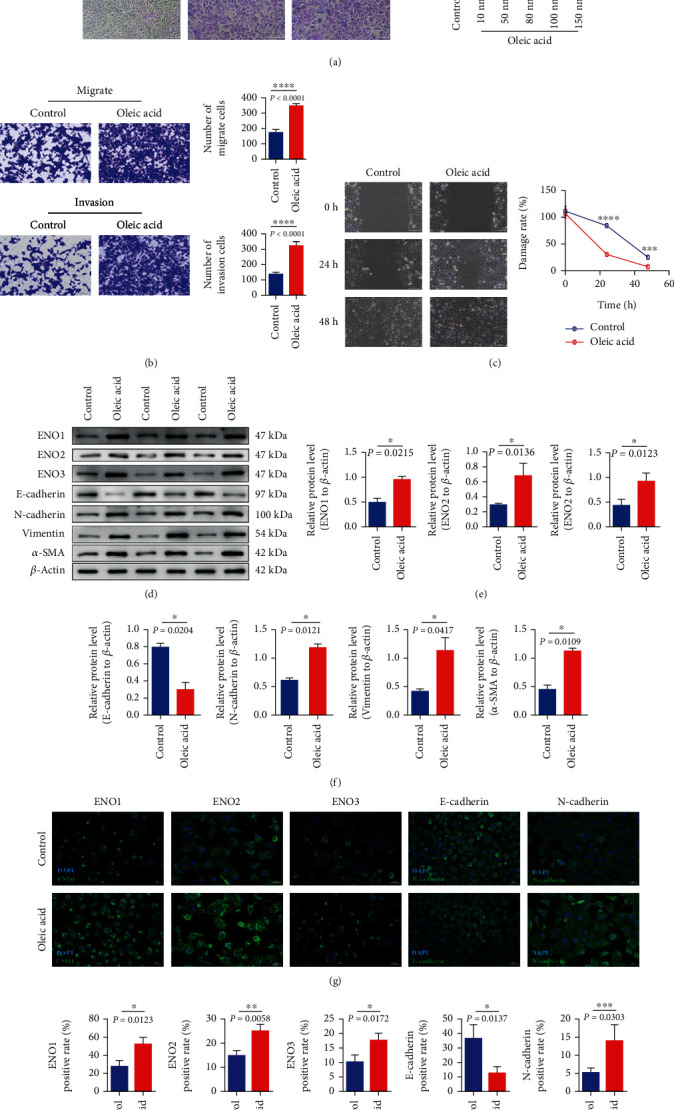
The cellular level confirmed that high-lipid upregulated enolase, accelerated the process of EMT, and aggravated the malignant behavior of pancreatic cancer cells. (a) PANC-1 cells were treated with different concentrations of oleic acid, and lipid formation was examined by oil Red O staining. (b, c) PANC-1 cells were stimulated with 100 nM oleic acid to detect cell migration and invasion. (d–f) WB was used to detect the expression of enolase-related indicators ENO1/ENO2/ENO3 and EMT-related indicators E-cadherin/N-cadherin/vimentin/*α*-SMA. (g, h) The expression of enolase-related indicators ENO1/ENO2/ENO3 was detected by immunofluorescence. Expression of EMT-related genes E-cadherin/N-cadherin.

Human pancreatic cancer cell PANC-1 high-fat cell model was established by oleic acid. It was confirmed that high-fat upregulated enolase accelerated the process of EMT and aggravated the malignant behavior of pancreatic cancer cells at the cellular level. To establish a high-fat cell model, PANC-1 cells were stimulated with oleic acid at concentrations of 10 nM, 50 nM, 80 nM, 100 nM, and 150 nM, respectively. After oil Red O staining, the number of oil Red O stained cells in the microscopic field increased with the increase of oleic acid concentration (Figure 6(a)). The results showed that the number of adipogenic cells increased with the increase of oleic acid concentration, and the percentage of adipogenic cells was higher when the concentration was 100 nM.

In order to explore the effect of high fat and hyperlipidemiaon on the migration and invasion of pancreatic cancer cells, a high-fat cell model was established, and the migration and invasion levels of cells were detected by transwell chamber assay. The results showed that the migration and invasion levels of cells in the high-fat group were enhanced compared with those in the control group (Figures 6(b) and 6(c)). These results indicated that high fat could promote the migration and invasion of pancreatic cancer cells. We detect the expression of enolase-related indicators ENO1/ENO2/ENO3 and EMT markers in high-fat cells and control cells, and further explore the effect of high fat on enolase level and migration ability of pancreatic cancer cells at the molecular level. Western blotting results showed that the enolase-related indicators ENO1/ENO2/ENO3 and the expression of EMT-related indexes N-cadherin, vimentin, and *α*-SMA of PANC-1 cells in the oleic acid intervention group were significantly higher than those in the control group (Figures 6(d)–6(f)). These results suggest that high fat promotes the expression of enolase and accelerates the process of EMT in pancreatic cancer cells.

In order to further clarify the effect of high fat on enolase-related indicators ENO1/ENO2/ENO3 and EMT markers of pancreatic cancer cells, immunofluorescence detection was performed. The results showed (Figures 6(g) and 6(h)) that the enolase-related indicators ENO1/ENO2/ENO3 of PANC-1 cells treated with oleic acid were significantly increased compared with the control group. The expression of EMT-related index N-cadherin in PANC-1 cells treated with oleic acid was higher than that in the control group, and the expression of E-cadherin in PANC-1 cells treated with oleic acid was lower than that in the control group. These results further suggested that, at the protein level, high fat increased enolase expression in pancreatic cancer cells accelerated EMT progression.

## 4. Discussion

The incidence of adenocarcinoma varies widely across regions, with lifestyle and environmental factors playing a significant role. Smoking is the most commonly known risk factor, and diabetes, high fat, chronic pancreatitis, and genetic mutations all contribute to an increased risk of pancreatic cancer [25]. ENO2 (also known as *γ*-enolase, neuron-specific enolase, and NSE) can catalyze the conversion of 2-phosphoglycerate to phosphoenolpyruvate in glycolysis [26]. It has two isoenzymes and is a very important enzyme in glycolysis. ENO2 is mainly found in neuronal cells and neuroendocrine cells [27]. Abnormal expression of ENO2 is associated with a variety of neurological injuries, which can be used as a marker to evaluate neuronal death in different CNS injuries [28]. ENO2 is highly expressed in tumor patients, especially in neurogenic and neuroendocrine tumors, and is considered to be the most important tumor marker of poorly differentiated neuroendocrine tumors. In addition, exposure to ENO2 in carcinogenic pollutants cadmium and arsenic can also be used as a marker [29]. Remodeling of actin cytoskeleton leads to cell migration. It has been found that ENO2 can bind actin and tubulin, thereby affecting microtubule motility and cell migration [30]. ENO2 depends on gamma-1-syntrophin to colocalize actin. *γ*-Enolase controls neuronal survival, differentiation, and neurite regeneration through the activation of PI3K/Akt and MAPK/ERK signaling pathways, thereby regulating cytoskeletal reorganization and cell remodeling [31]. RhoA inhibits axon elongation, while ENO2 inactivates RhoA through PI3K [32]. By analyzing the clinical-related indexes of pancreatic cancer patients and the control group, this study found that the triglyceride level of pancreatic cancer patients was higher than that of the control group at the initial diagnosis [33]. The level of neuron-specific enolase (NSE) in pancreatic cancer patients was higher than that in the control group [3]. Triglyceride level was positively correlated with NSE. It is suggested that triglyceride is a warning indicator of pancreatic cancer [34]. Blood lipid analysis combined with ENO2 analysis is helpful to evaluate the condition of pancreatic cancer patients.

Triglycerides, an important component of blood lipids, were found to be more likely to develop lung cancer in people with hypertriglyceridemia in an Austrian study [35]. Other studies have confirmed that the lipid content of some dendritic cells (DC) is increased, especially the triglyceride content [36]. There should be a high level of lipids, especially triglycerides, in tumor cells, which further stimulates and promotes the expression of ENO1 [37]. A long-term high-fat diet is an important contributor to obesity, which increases the risk of pancreatic cancer. In this study, it was found that the tumor volume of mice in the high-fat group increased faster in the subcutaneous tumorigenesis test, and the lung metastasis of mice in the high-fat group was more frequent in the tail vein injection [38]. Triglyceride storage in vitro, lipid droplet formation, and lipid accumulation in vivo of mouse pancreatic cancer cells can positively regulate tumor growth [39]. HE staining was used to observe the lesion morphology. PCR, WB, and immunohistochemistry were used to detect ENO1, ENO2, ENO3, and EMT indicators (E-cadherin, N-cadherin, and vimentin). It was found that enolase increased significantly in the high-fat group, and EMT indicators also showed a positive effect. These results indicated that high fat could increase enolase, promote EMT, and accelerate the development of pancreatic cancer in mice. Triglycerides exist in peripheral blood in pancreatic cancer cells in large quantities, which leads to the increase of triglyceride level in the circulating blood of patients [40]. The high concentration of triglyceride in the pancreas and peripancreas can hydrolyze pancreatic enzymes and produce a large amount of free fatty acids locally to induce acidosis, which can activate trypsinogen, thus triggering a series of activation of trypsinogen and then leading to severe self-digestion of the pancreas. High levels of triglyceride can damage vascular endothelium and increase blood viscosity. In severe pancreatitis, a large number of plasma components seep out and blood is concentrated under the action of various inflammatory factors. In this study, oleic acid was used to construct a high-fat pancreatic cancer cell model (PANC-1 cell line) to detect the biological behavior of pancreatic cancer cells after an intervention. It was found that the expressions of ENO1, ENO2, and ENO3 of PANC-1 cells were increased under high-fat conditions, and the migration and invasion ability of cancer cells was enhanced. Immunofluorescence detection of pancreatic cancer cells in the high-fat group and the control group showed that the trend of EMT-related indicators was consistent with that of the animal model, suggesting that high fat promoted the process of EMT. The trend is consistent; in in vivo and in vitro experimental results, it is concluded that high cholesterol and obesity caused by high-fat diet, which degrades the pancreas, induces inflammation, promotes the enolization enzyme expression, increases the risk of pancreatic cancer, and accelerates the progress of pancreatic cancer; low-fat diet helps pancreatic cancer prevention but also for the future further research provides the basis for pancreatic cancer.

## 5. Conclusion

In in vivo experiments, mouse models of subcutaneous tumorigenesis and metastasis of pancreatic cancer cells injected into the tail vein were constructed. In in vitro cell experiments, PANC-1 cells were intervened with oleic acid to construct a high-fat cell model. Enolase-related indicators ENO1/ENO2/ENO3 and EMT-related indicators were detected. The hyperlipidemia mouse model was established, and the hyperlipidemia animal model was successfully established by lipid four items and oil Red O staining. Subcutaneous tumorigenesis of pancreatic cancer cells in the hyperlipidemia group and control group showed that hyperlipidemia promoted tumor growth. Enolase-related indicators ENO1/ENO2/ENO3 and EMT-related indicators E-cadherin/N-cadherin/vimentin/*α*-SMA were detected by qPCR, western blotting, and immunohistochemistry. It was found that hyperlipidemia could increase the expression of enolase-related indicators ENO1/ENO2/ENO3 in subcutaneous tumorigenesis tissues and accelerate the process of EMT. The results of tail vein injection of pancreatic cancer cells in the hyperlipidemia group and control group showed that hyperlipidemia increased the expression of enolase-related indicators ENO1/ENO2/ENO3 in metastatic tumor tissues, accelerated the process of EMT, and promoted the metastasis of pancreatic cancer cells. PANC-1 cells were treated with oleic acid to establish a high-lipid cell model. With the increase of oleic acid concentration, cell adipogenesis was enhanced. After oleic acid intervention in PANC-1 cells to establish a high-lipid cell model, it was confirmed at the cell level that high lipid enhanced the migration and invasion ability of pancreatic cancer cells by increasing the expression of enolase-related indicators ENO1/ENO2/ENO3.

## Data Availability

All data generated or analyzed during this study are included in this article.

## Abbreviations

ADM: Acinar-to-ductal metaplasia
CCK8: Cell Counting Kit-8
DAB: Diaminobenzidine
ECL: Electrochemiluminescence
EMT: Epithelial-mesenchymal transition
ENO: Alpha-enolase
FAK: Focal adhesion kinase
GAL-3: Galactase-3
HDL-C: High-density lipoprotein
HE: Hematoxylin-eosin
HTG: Hypertriglyceridemia
HTGP: HTG-induced pancreatitis
IS: Ischemic stroke
LDL-C: Low-density lipoprotein
NSE: Neuron-specific enolase
OD: Optical density
PANIN: Pancreatic intraepithelial neoplasia
PDAC: Pancreatic adenocarcinoma
PI: Propidium iodide
PTL: Pancreatic triglyceride lipase
ROC: Receiver operating characteristic
ROCK: Rho-associated kinase
SCR: Serum creatinine.
